# A lesion in two: Buruli ulcer and squamous cell carcinoma coexistence

**DOI:** 10.1371/journal.pntd.0011911

**Published:** 2024-02-08

**Authors:** Jessica C. O’Keeffe, Albert H. Yin, Daniel P. O’Brien

**Affiliations:** 1 Department of Infectious Diseases, Barwon Health, Geelong, Victoria, Australia; 2 Barwon South West Public Health Unit, Geelong, Victoria, Australia; 3 Department of Anatomical Pathology, The Royal Melbourne Hospital, Parkville, Victoria, Australia; University of Connecticut, UNITED STATES

## Abstract

The concurrent diagnoses of Buruli ulcer (BU) and cutaneous squamous cell carcinoma (SCC) is a phenomenon not previously described, despite the fact that both conditions are highly prevalent in Australia. This report presents an intriguing case of concurrent diagnoses, with clues alluding to more than one skin condition being present. The case involves a 73-year-old man with BU diagnosed on the scalp, an atypical location, which led to the consideration of malignancy, ultimately revealing concurrent SCC. This case highlights the importance of considering both conditions in patients with epidemiological risk factors, necessitating multiple lines of investigation for accurate diagnosis. Medical practitioners must remain vigilant and incorporate this possibility into their diagnostic algorithms for suspicious skin lesions to optimize treatment and outcomes. This is the first recorded instance of simultaneous diagnosis, underlining the need for enhanced awareness and attention to these unique cases.

## Author summary

- Buruli ulcer (BU) and squamous cell carcinoma (SCC) can coexist in the same lesion.
- SCC and other cutaneous malignancies may act as a portal of entry for the mycobacteria.
- Clinical differentiation is challenging, and biopsy should be performed, with tissue sent for histopathological evaluation as well as *Mycobacterium ulcerans* PCR.
- Consider the possibility of underlying malignancy if a *Mycobacterium ulcerans* lesion occurs in an unusual body site (for example: on the scalp) or does not heal as expected, despite appropriate treatment.

## Introduction

In the realm of ulcerative skin conditions, the coexistence of distinct diseases can present intriguing challenges for diagnosis and treatment. This case report explores a situation where Buruli ulcer (BU) and cutaneous squamous cell carcinoma (SCC) were diagnosed concurrently, an unreported occurrence that has important implications for medical practitioners. Buruli ulcer, caused by *Mycobacterium ulcerans* (*M*. *ulcerans*), leads to progressive destruction of the skin and soft tissues, while SCC arises from cells in the stratum spinosum layer of the epidermis. Though previously described as a long-term complication of BU, typically presenting in the scar of a previous ulcer more than 10 years post original infection [1–3], the previously unreported concurrent diagnoses of these conditions poses unique diagnostic complexities due to shared clinical features.

### Clinical record

**Fig 1:**
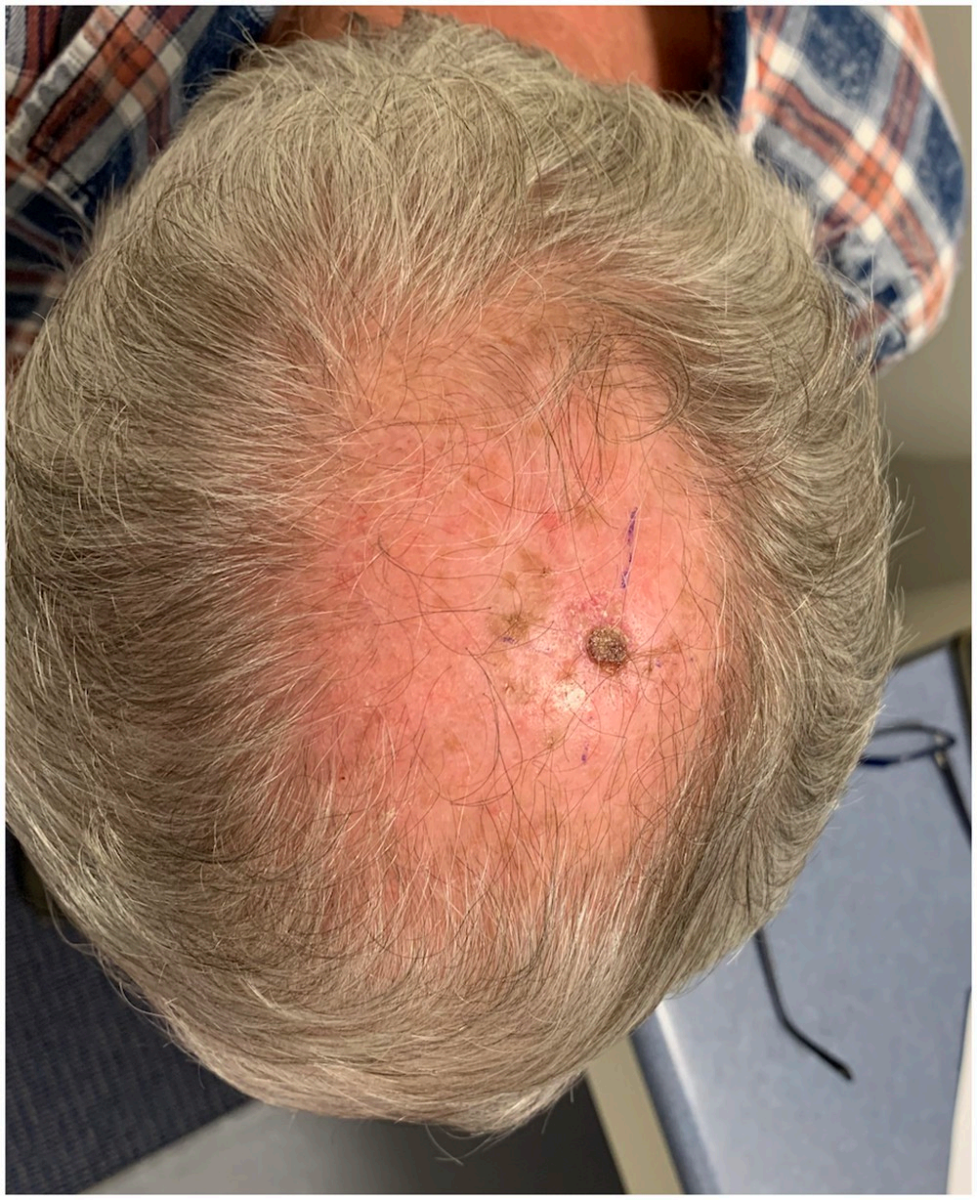
Initial painful ulcerative scalp lesion measuring 20 × 15 millimetres (mm) (induration) and 6 × 6 mm (ulceration).

**Fig 2:**
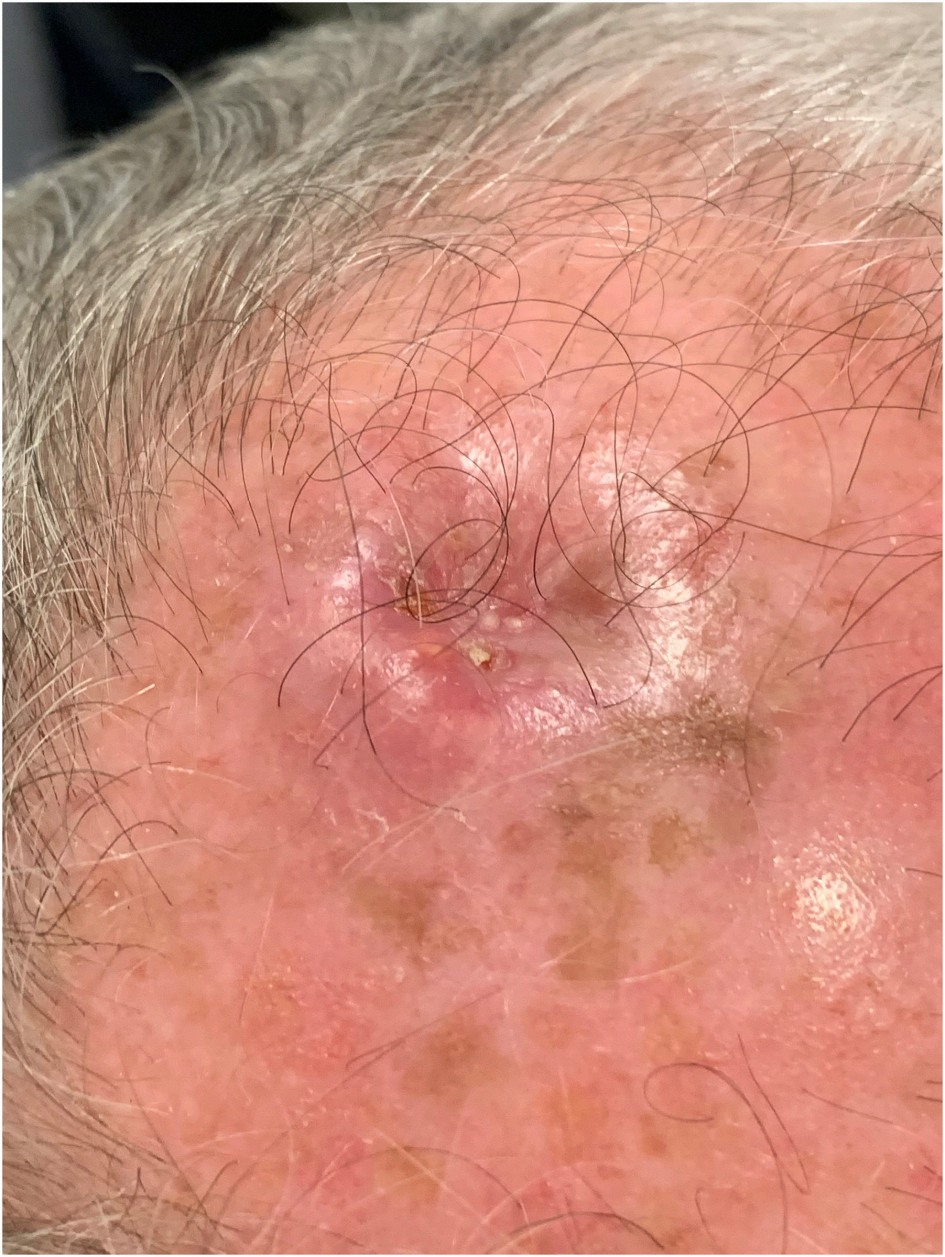
Ongoing scalp lesion at 8 months post commencement of antimicrobial therapy.

**Fig 3:**
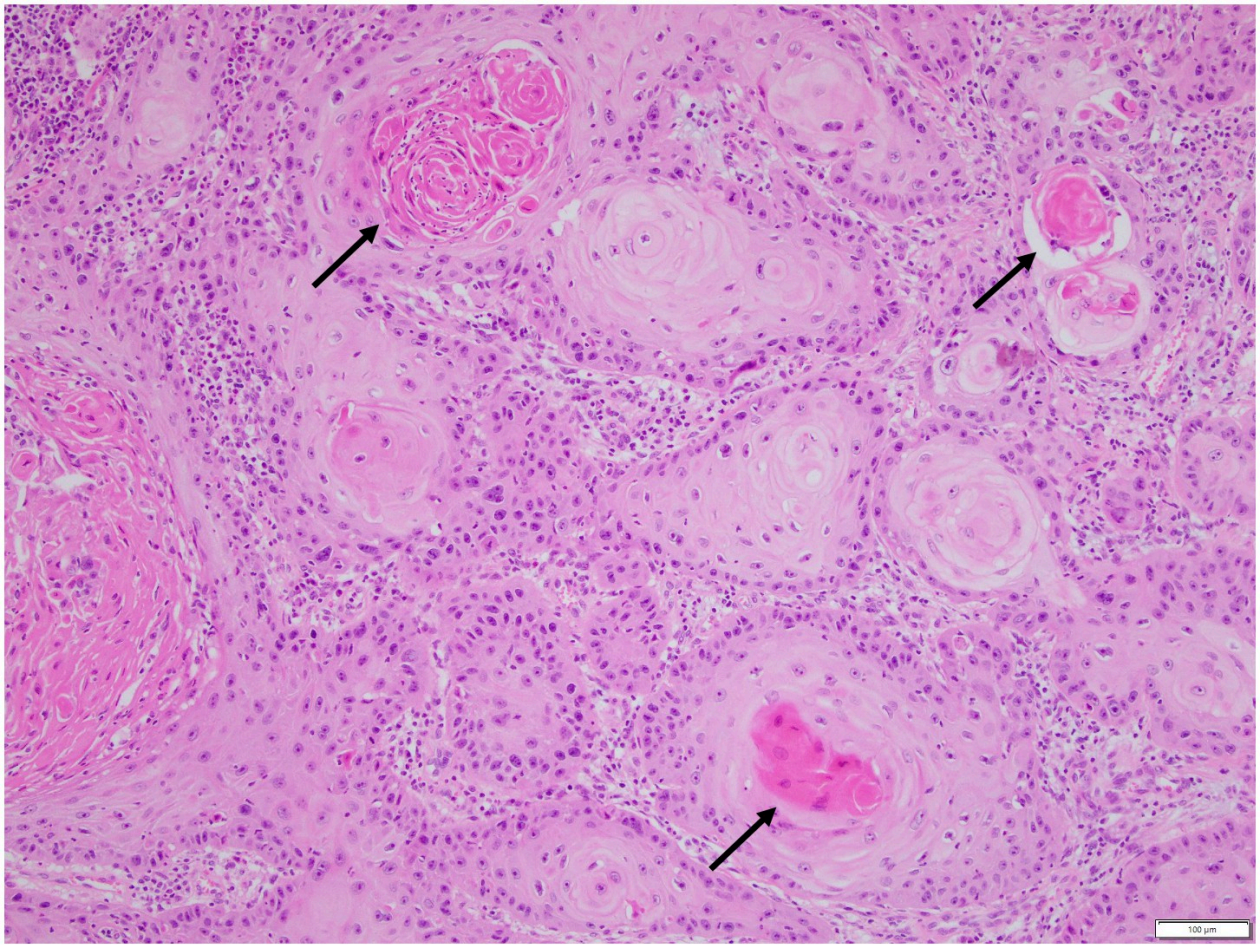
Squamous cell carcinoma with keratin pearl formation (arrows).

**Fig 4:**
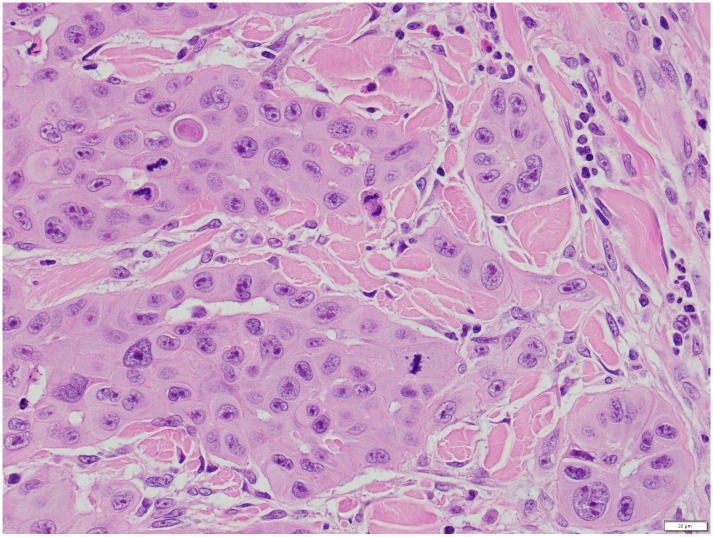
Marked nuclear atypia and mitotic activity.

A 73-year-old retired man from the south eastern coastal region of Australia with no significant past medical history presented with 2 months of a painful ulcerative lesion on his scalp measuring 20 × 15 millimetres (mm) (induration) and 6 × 6 mm (ulceration) (Fig 1). *M*. *ulcerans* was diagnosed on polymerase chain reaction (PCR) performed on a superficial swab of the ulcer (cycle threshold values of 32 and 32 on 2 runs, culture negative at 12 weeks). He completed systemic treatment with 8 weeks of Rifampicin 300 milligrams (mg) twice daily and Clarithromycin 500 mg twice daily without adverse events, while local treatment included standard wound care. The lesion never entirely healed despite its small size on presentation (World Health Organization Category I) (Fig 2). The beginning of skin healing over the lesion occurred after 6 months compared with the median 3 months for similar lesions [4], and the skin over the area of initial infection remained irregular with some regression of healing over the following 2 months. Subsequent biopsy of the region demonstrated SCC and he was referred for plastic surgical management involving a wide excision and split skin graft (see Figs 3 and 4). No *M*. *ulcerans* recurrence was detected after 12 months follow-up since commencement of treatment.

## Discussion

BU and cutaneous SCC are distinct skin conditions that we have described occurring simultaneously, though SCC has only previously been described as a long-term complication of BU, [5]. Buruli ulcer is a chronic, necrotizing skin disease caused by *M*. *ulcerans*, which results in progressive destruction of the skin and soft tissues, and rarely, the underlying bone [6]. Squamous cell carcinoma is a cancer arising from the stratum spinosum layer of the epidermis [7]. Despite their different etiologies, these 2 skin conditions share clinical features that can make diagnosis challenging [5,6]. It is plausible that the loss of integrity of the skin affected by malignant cells may act as a portal of entry, leading to a secondary *M*. *ulcerans* infection [6]. *M*. *ulcerans* is endemic in Australia [8,9], and SCC is common in Australian populations; therefore, it is important that medical practitioners are aware of the potential coexistence of these conditions.

A clue to the coexistence in this case was the presentation in a highly atypical location on the body. BU generally occurs on the limbs as highlighted in a retrospective observational study of 649 people from the same endemic area as our case, where none of the lesions occurred on the scalp [9]. Given that this location is much more commonly affected by cutaneous malignancies, clinical suspicion for malignancy was high from the time of presentation. Although it is possible the *M*. *ulcerans* PCR was a false positive, this risk is low [10] given the cycle threshold value of 32 on 2 separate samples, and the clinical response to treatment with initial healing of the skin over the lesion suggesting that it was a true positive result. Although the swab was culture negative for *M*. *ulcerans*, this is not unusual due to the low culture sensitivity of this method [8].

The importance of histopathologic analysis is also highlighted here, and its utility in diagnosing not only cutaneous malignancies, but also mycobacterial infection. In this case, *M*. *ulcerans* PCR was positive, though diagnosis can be made in those from endemic regions with clinically consistent lesions and histopathology showing acid fast bacilli, even in the absence of PCR [8].

To our knowledge, this is the first description of BU and SCC diagnosed simultaneously, and it has important implications for diagnosis, treatment, and management of these diseases. Therefore, it is essential that medical practitioners remain vigilant and alert to this possibility and consider it in their diagnostic algorithms for suspicious lesions, especially if in atypical parts of the body, by ordering histology including acid fast bacilli stains and *M*. *ulcerans* PCR on biopsy specimens in patients who have epidemiological risk factors for concurrent diagnoses.
